# A Novel Mouse Model for Multiple Myeloma (MOPC315.BM) That Allows Noninvasive Spatiotemporal Detection of Osteolytic Disease

**DOI:** 10.1371/journal.pone.0051892

**Published:** 2012-12-20

**Authors:** Peter O. Hofgaard, Henriette C. Jodal, Kurt Bommert, Bertrand Huard, Jo Caers, Harald Carlsen, Rolf Schwarzer, Nicole Schünemann, Franziska Jundt, Mona M. Lindeberg, Bjarne Bogen

**Affiliations:** 1 Centre for Immune Regulation, Institute of Immunology, University of Oslo and Oslo University Hospital, Oslo, Norway; 2 Comprehensive Cancer Centre Mainfranken and Department of Internal Medicine II, Division of Haematology and Medical Oncology, University Hospital Würzburg, Würzburg, Germany; 3 Division of Hematology, University Hospitals of Geneva and Department of Pathology-Immunology, Geneva University Medical Centre, Geneva, Switzerland; 4 Department of Hematology, University of Liège, Liège, Belgium; 5 Institute for Nutrition Research, University of Oslo, Oslo, Norway; 6 Department of Hematology, Oncology, and Tumor Immunology, Charité-Universitätsmedizin, Campus Virchow Clinic, Molekulares Krebsforschungszentrum, Berlin, Germany; 7 Max-Delbrück-Center for Molecular Medicine, Berlin, Germany; University of Campinas, Brazil

## Abstract

Multiple myeloma (MM) is a lethal human cancer characterized by a clonal expansion of malignant plasma cells in bone marrow. Mouse models of human MM are technically challenging and do not always recapitulate human disease. Therefore, new mouse models for MM are needed. Mineral-oil induced plasmacytomas (MOPC) develop in the peritoneal cavity of oil-injected BALB/c mice. However, MOPC typically grow extramedullary and are considered poor models of human MM. Here we describe an *in vivo*-selected MOPC315 variant, called MOPC315.BM, which can be maintained *in vitro*. When injected i.v. into BALB/c mice, MOPC315.BM cells exhibit tropism for bone marrow. As few as 10^4^ MOPC315.BM cells injected i.v. induced paraplegia, a sign of spinal cord compression, in all mice within 3–4 weeks. MOPC315.BM cells were stably transfected with either firefly luciferase (MOPC315.BM.Luc) or DsRed (MOPC315.BM.DsRed) for studies using noninvasive imaging. MOPC315.BM.Luc cells were detected in the tibiofemoral region already 1 hour after i.v. injection. Bone foci developed progressively, and as of day 5, MM cells were detected in multiple sites in the axial skeleton. Additionally, the spleen (a hematopoietic organ in the mouse) was invariably affected. Luminescent signals correlated with serum myeloma protein concentration, allowing for easy tracking of tumor load with noninvasive imaging. Affected mice developed osteolytic lesions. The MOPC315.BM model employs a common strain of immunocompetent mice (BALB/c) and replicates many characteristics of human MM. The model should be suitable for studies of bone marrow tropism, development of osteolytic lesions, drug testing, and immunotherapy in MM.

## Introduction

Recent progress in unraveling the biology of MM, particularly the intracellular signaling pathways and the interactions with the bone marrow microenvironment, has resulted in development of novel targeted therapy [1]. Different animal models for MM disease have contributed to this progress, each model having advantages and disadvantages in this respect.

Xenograft models using human myeloma cell lines, or primary human myeloma have been established in immunodeficient SCID or NOD scid gamma (NSG) mice[2]–[8]. In particular, models have been generated where human MM cells grow in human fetal bone transplants in immunodeficient SCID mice [9]. More recently three-dimensional bone-like scaffolds were coated with mouse or human bone marrow stromal cells and implanted under the skin of SCID mice. Subsequently, injection of purified primary myeloma cells into these scaffolds gave rise to tumor formation that could be followed by measuring myeloma protein concentration [10]. Although these models allow experiments of human MM cells *in vivo* in mice, the models are demanding and not completely physiological.

Mouse models where MM cells can be transferred between syngeneic mice are also available. However, mouse MM models do not necessarily accurately reflect human disease. MM-like disease arises spontaneously in aged C57BL/KaLwRij mice[11]–[13]. The 5T2MM and the 5T33MM cell lines were established from such mice, and have been extensively used for studying homing mechanisms of MM cells to bone marrow, interaction of MM cells with the bone marrow environment, and evaluation of new therapies [14]. Both models are characterized by MM cell infiltration restricted to bone marrow and spleen (a hematopoietic organ in mice) [15]. The 5T2MM model, but not 5T33MM, is associated with an extensive osteolysis, seen on plain radiographs of femur and tibia[15]–[16]. Both 5T2MM and the 5T33MM are maintained by *in vivo* passages and cells die after 24 to 48h in culture. However, two *in vitro* growing subclones of the 5T33MM model have been developed, namely the 5T33MM*vt* [17] and the 5TGM1 cells [18]. Intravenous injection of 5TGM1 cells give rise to MM-like osteolytic disease. 5TGM1 cells have also been labeled with luciferase [19] and green fluorescent protein (GFP) [20], and used for *in vivo* imaging of MM-like disease. A major drawback of the 5TMM models is the dependency on a particular mouse strain (C57BL/KaLwRij) of limited availability.

Finally, three different transgenic mice models have recently been developed based on double-transgenic Myc/Bcl-XL mice [21], the activation of MYC under the control of a light chain gene [22], or cloning of a spliced form of mouse XBP-1 downstream of the immunoglobulin VH promoter and enhancer elements [23]. Although they recapitulate several characteristics of MM, these models are time-consuming and costly, perhaps explaining their limited use thus far.

In summary, the available MM models presented above can be technically challenging and require large investments. Thus, there is a need for an MM model where MM cells can be grown *in vitro* and when i.v. injected in a common laboratory inbred mouse strain, such as BALB/c, faithfully duplicate the major characteristics of MM disease seen in patients.

Plasmacytomas can be experimentally induced in certain strains of mice by i.p. injection of mineral oil, adjuvants and alkanes [24]. Such mineral oil-induced plasmacytomas (MOPC) can be serially transplanted s.c. or i.p. and have been extensively used in tumor immunological studies[25]–[30]. However, these plasmacytomas typically grow locally at the site of injection, and only infrequently metastasize to the bone marrow[31]–[33]. Due to their local growth, it has been questioned if MOPC tumors represent good models for human MM that primarily affects bone marrow.

We have previously described an *in vivo*-selected variant of MOPC315, MOPC315.4, which efficiently forms local tumors after s.c. injection [34]. We here show that repeated i.v. injections of MOPC315.4 cells, followed by isolation of tumor cells from femurs between passages, enriches for a stable variant (MOPC315.BM) that can be grown *in vitro*, has tropism for bone marrow after i.v. injection, and causes osteolytic lesions. Spatiotemporal development of disease may be monitored by serial and noninvasive measurement of the bioluminescent signal of luciferase-labeled cells.

## Results

### A Multiple Myeloma-like Cell Line, MOPC315.BM, which Homes to and Expands in the Bone Marrow after i.v. Injection

**Figure 1:**
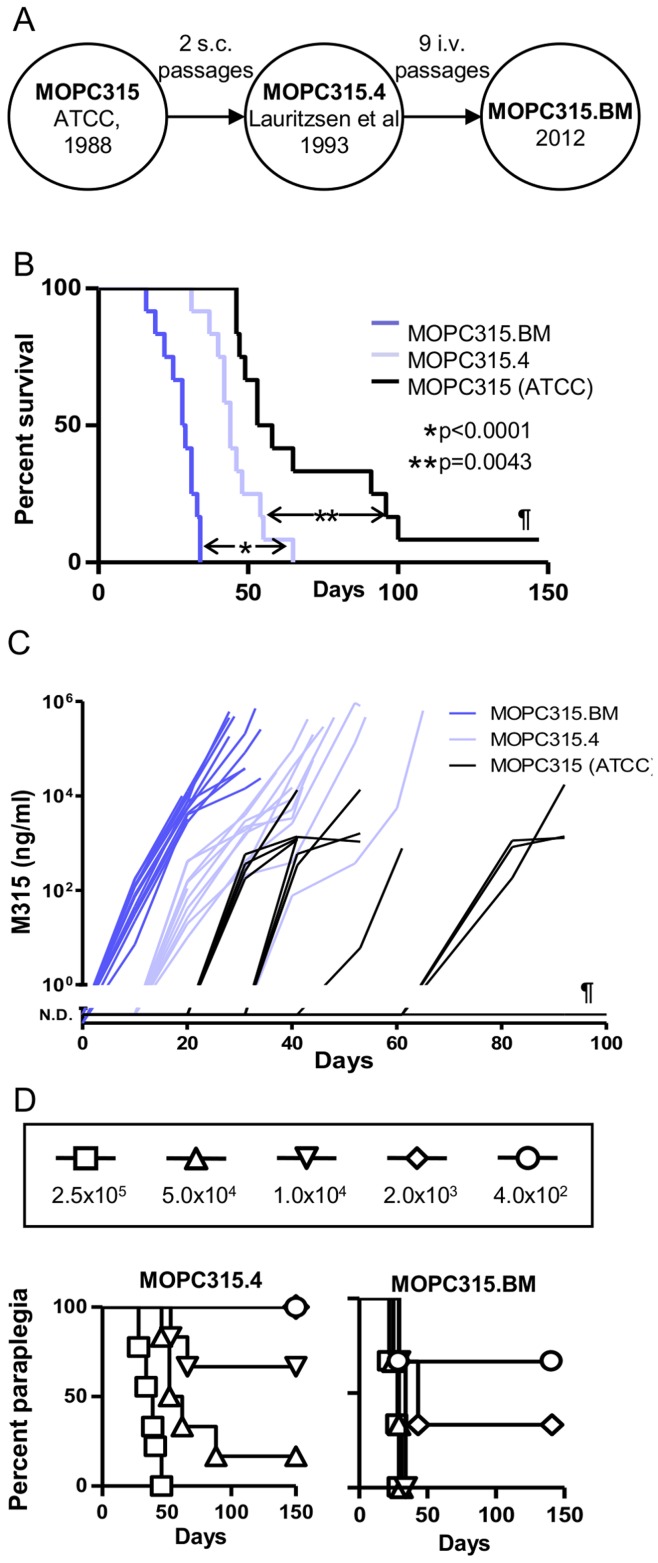
The development and tumorigenicity of MOPC315.BM. (A) The historic line of development of MOPC315.BM in senior author’s lab. (B) BALB/c mice were injected i.v. with MOPC315.BM, MOPC315.4 or MOPC315 (ATCC) (2×10^5^, n = 12/group). All mice developed at the endpoint paraplegia due to spinal cord compression except two mice injected with MOPC315 (ATCC) that reached the endpoint by weight loss. One mouse (¶) injected with MOPC315 (ATCC) did not develop tumor disease at all. The survival curves are significantly different, both for MOPC315.BM compared to MOPC315.4 (*p<0.0001) and MOPC315.4 compared to MOPC315 (ATCC) (**p = 0.0043). (C) Serum myeloma protein M315. At day 10, M315 levels for MOPC315.BM were significantly different from MOPC315 and MOCP315.4 (p<0.0001). At day 20 and 30, M315 levels for all three cell lines were significantly different from each other (p<0.003). One mouse (¶) did not develop detectable levels of M315, this being the same mouse that did not develop tumor disease as shown in A. Data are presented as mean ± SD in Fig. S1. N.D. = Not Detected. (D) Titration of MOPC315.BM and MOPC315.4 by i.v. injection and development of paraplegia (n = 3–6/group).

Previously, an *in vitro*-adapted MOPC315 cell line from ATCC (TIB23) was passaged s.c. twice to obtain a variant, MOPC315.4, that consistently produced s.c. extramedullary plasmacytomas [34]. This cell line has been extensively used in tumor immunological studies[26]–[30]. To obtain a MM-like variant cell line with tropism for the bone marrow, MOPC315.4 cells were injected i.v. at a high dosage (2×10^6^). Tumor cells were flushed from femurs of the first mouse that developed paraplegia, a sign of spinal cord compression. This procedure was repeated 9 times (Fig. 1A). The final cell line, MOPC315.BM, was compared with MOPC315.4 and MOPC315 (ATCC) in tumor challenge experiments in which the same number of *in vitro*-cultured cells (2×10^5^) were injected i.v. (Fig. 1B). Tumor development was monitored by serial measurements of M315 myeloma protein in serum (Fig. 1C, Fig. S1A). The endpoints were defined as 1) paraplegia, 2) visible extramedullary tumor growth with tumor size >1 cm in diameter, 3) distended abdomens, or 4) weight loss >10%.

All mice injected with MOPC315.BM cells developed paraplegia within 30 days. Paraplegia was also induced by MOPC315.4, but disease development was delayed by about 14 days compared to MOPC315.BM. The ATCC line was slowest at causing spinal involvement, moreover, 2 out of 12 mice died of tumor disease but without paraplegia, and one mouse did not develop tumor disease at all (Fig. 1B). Consistent with these results, mice injected with MOPC315.BM cells had a more rapid increase in serum M315 myeloma protein compared with the other two cell lines (Fig. 1C, Fig. S1A). Notably, the increase in serum M315 myeloma protein is more uniform in mice injected with MOPC315.BM, than mice injected with the MOPC315.4 or the MOPC315 (ATCC) cell lines. These results establish a hierarchy for the rapidity of spinal MM disease onset: MOPC315.BM>MOPC315.4>MOPC315 (ATCC).

Next, titrated amounts of MOPC315.BM and MOPC315.4 cells were injected i.v. (Fig. 1D). The results show that the MOPC315.BM line was more tumorigenic than parental MOPC315.4 by a factor of ∼20. As few as 2000 or 400 MOPC315.BM cells caused paraplegia in respectively 66% and 33% of the mice within 50 days, while as many as 50,000 MOPC315.4 cells were required to cause paraplegia in 17% of mice within the same time frame. This finding indicates that an increased fraction of MOPC315.BM cells have the ability to establish progressive bone marrow disease after i.v. injection, compared to MOPC315.4.

Given that the MOPC315 cell line after i.v. injection appears to induce MM-like disease, we tested the sensitivity of MOPC315.BM to the MM drug bortezomib, a proteasome inhibitor, *in vitro* (Fig. S2). The IC50 was 10–15 nM, which is in the same range as that previously reported for chemosensitive MM cell lines [35].

### Tagging MOPC315.BM Cells with Luciferase Produced a Cell Line (MOPC315.BM.Luc), with a Delayed Onset of Paraplegic Disease

**Figure 2:**
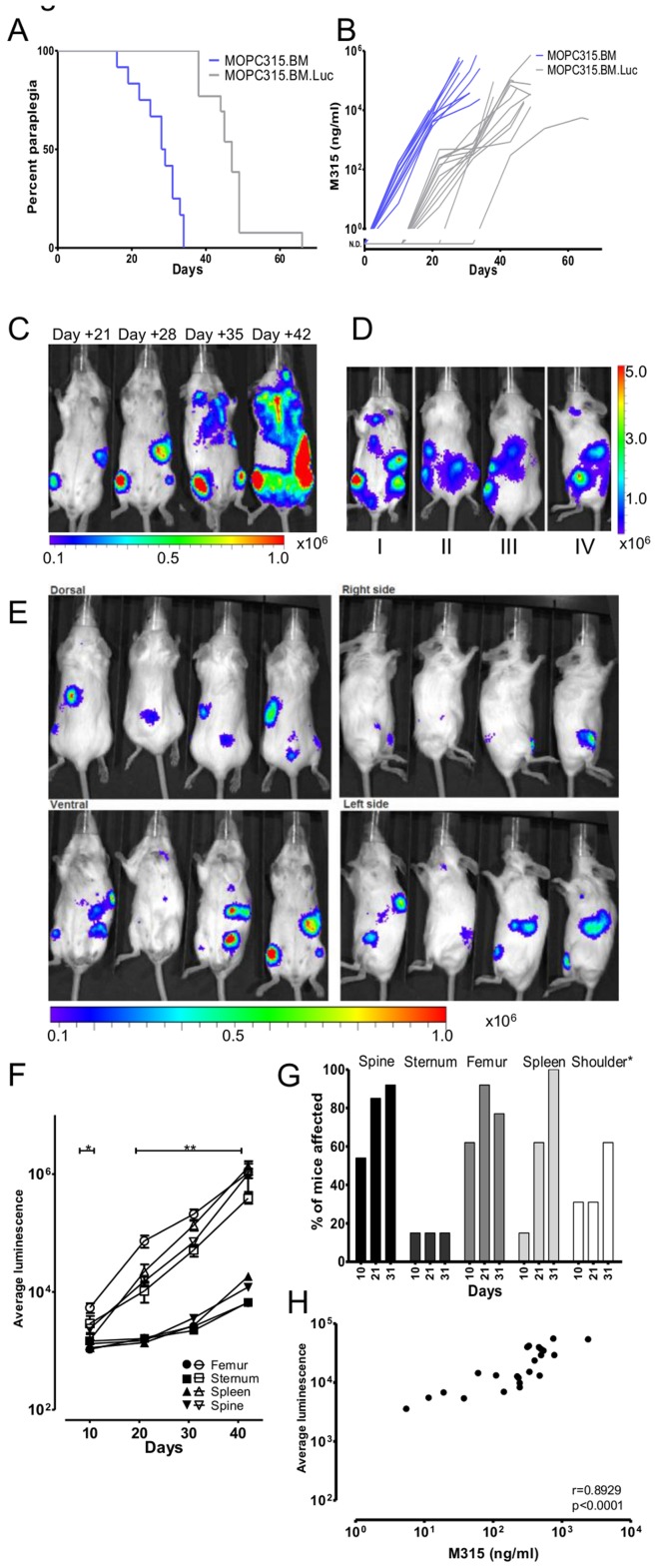
Delayed growth and BLI of Luciferase-transfected MOPC315.BM-cells. (A) Tumor take experiment with MOPC315.BM.Luc (2×10^5^ cells i.v.) (n = 13) was overlaid that of MOPC315.BM shown in Fig. 1B (also 2×10^5^ cells i.v.). The survival curves showing development of paraplegia are significantly different (p<0.0001). (B) Levels of M315 myeloma protein in sera of mice in (A). M315 was significantly different at day 10, 20 and 30 (p<0.002). N.D. = Not Detected. (C–E) All scales in photons/second/cm^2^/steradian. (C) Timeline of bioluminescent signals in a representative BALB/c mouse injected with 2.0×10^5^ MOPC315.BM.Luc i.v. (D) Examples of typical sites of affection. I: sternum, femurs, spleen. II, III: spine, femurs, spleen. IV: spine, femurs, spleen, shoulder. (E) Tumor growth of MOPC315.BM.Luc in four representative BALB/c mice pictured at day 28. (F) Luminescence emitted from typical sites of growth as a function of time (n = 13). MOPC315.BM.Luc mice (open boxes) are compared to non-injected control mice (filled boxes). *p<0.031 for femur and spine. **p<0.04 for all indicated sites. (G) Frequency of tumor growth in various sites on day 10, 21 and 31. *Signals from the shoulder region could not be attributed to particular bones. (H) Correlation of serum myeloma protein M315 measured on days 10, 21 and 32 and average luminescence [radiance (photons/second/cm^2^/steradian)] emitted in individual mice (r = 0.8929, p<0.001).

MOPC315.BM cells were transfected for constitutive expression of the firefly luciferase gene (MOPC315.BM.Luc). We first tested if transfection affected tumor growth. The MOPC315.BM.Luc cells were considerably slower at inducing paraplegic disease than the untransfected counterpart (Fig. 2A). This was confirmed by a delayed increase in serum myeloma protein (Fig. 2B, Fig. S1B). This finding prompted us to investigate if growth and myeloma protein secretion differed for the various cell lines *in vitro* (Table S1). The results show that MOPC315.BM and the MOPC315.BM.Luc cells grow at nearly equal rates and faster than both MOPC315 (ATCC) and MOPC315.4. For unknown reasons, MOPC315.BM.Luc secreted higher amounts of M315 myeloma protein than the other three cell lines. Based on these results, it appears likely, but not proven, that the slow kinetics of disease development of MOPC315.BM.Luc compared to MOPC315.BM in BALB/c mice is due to a low level immunogenicity to luciferase, resulting in slower growth but not elimination of luciferase-marked cells.

### Spatiotemporal Monitoring of MM Disease using MOPC315.BM.Luc Cells by Bioluminescence Imaging (BLI)

In MOPC315.BM.Luc-injected mice, tumor burden and localization could be detected by use of a CCD camera (IVIS Spectrum), as previously described in other tumor models [36]. Repetition of this noninvasive procedure throughout the course of the experiment resulted in a spatiotemporal resolution of tumor development in single animals.

When 2×10^5^ cells were injected in BALB/c mice, signals were detected by day 10 and increased progressively (Fig. 2C,E,F). With time, signals from individual foci (Fig. 2C) and the overall signals (Fig. 2F) increased in strength, and an increasing number of foci could be detected (Fig. 2C,G). Typical sites of bone affection were spine = femur>shoulder region>sternum (Fig. 2D,G). In addition, signals were frequently seen corresponding to the hip region, but in BALB/c mice the level of resolution was not sufficient to pinpoint localization. Signals from the spleen were present in 100% of mice (Fig. 2G), while signals from other internal organs including liver were rarely detected.

### Bioluminescence is a Valid Measurement for Tumor Load in BALB/c Mice

In the experiment of Fig. 2, both luminescence and serum myeloma protein were followed over the course of the experiment. The results show that the bioluminescence signals correlated with M315 serum concentrations in single mice (Fig. 2H, r = 0.8929, p<0.001). Moreover, the two methods for assessment of tumor load have approximately the same sensitivity although the data indicate that measurement of myeloma protein in an idiotype-specific ELISA is slightly more sensitive. A significant bioluminescent signal was definitely reached when myeloma protein concentration exceeded 100 ng/ml. Because serum myeloma protein concentration is considered to be a gold standard for tumor load in MM, one may conclude that the strength of the bioluminescence signal is a valid measurement for tumor size.

### BLI and CT of BALB/c nu/nu Mice: Monitoring Tumor Growth and Localization to the Axial Skeleton

**Figure 3:**
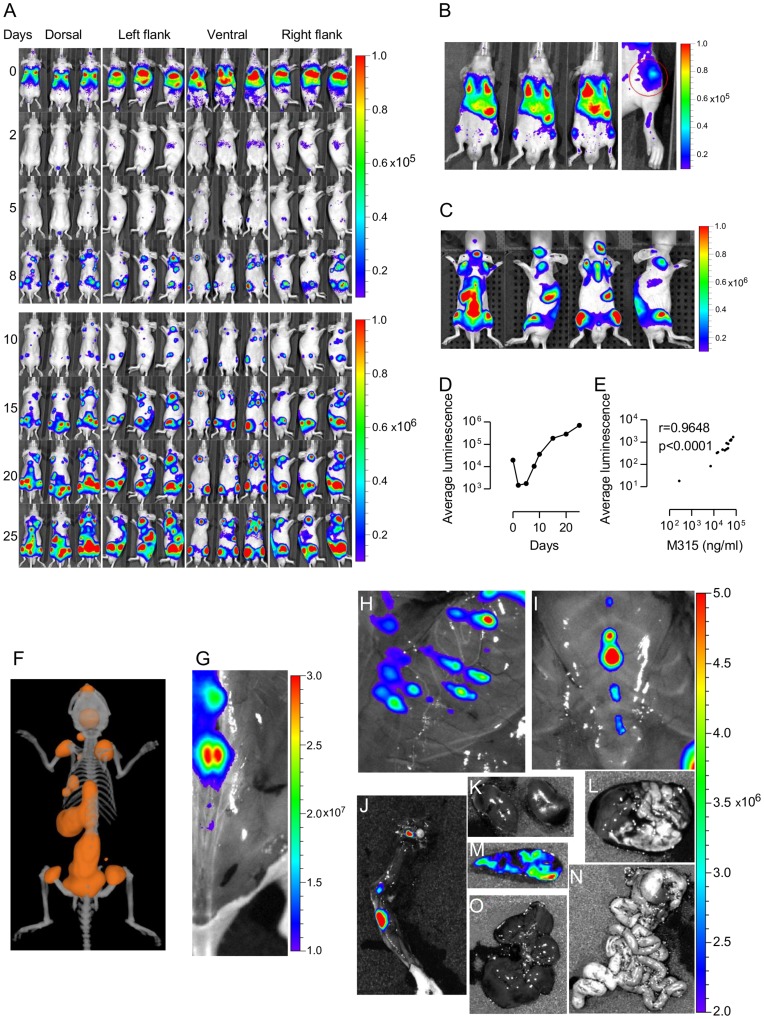
Co-localization of tumor cells and axial skeleton in MOPC315.BM.Luc injected in BALB/c nu/nu mice. (A) A photomontage of three representative BALB/c nu/nu mice (out of 14) serially imaged from day 0 to day 25. For days 0–8, the color scale was set at a radiance (photons/second/cm^2^/steradian) of 10^4^ to10^5^ (F stop 1, exposure 60 seconds or autoexposure 10^4^ counts, binning 8). From day 10 to 25 the scale was set at a radiance of 10^5^ to10^6^. (F stop 1, exposure 60 seconds or autoexposure 10^4^ counts, binning 1). (B, left picture) Ventral view of 3 mice injected 1h previously, showing weak signals emanating from the tibiofemoral regions. Such signals were consistently detected in 14/14 mice 1–2.5h after i.v. injection. Color scale was set at a radiance (photons/second/cm^2^/steradian) of 10^4^ to10^5^. (B, right picture) A close-up of the region of interest indicated by the red circle. (C) A close-up picture series of one mouse (day 33) showing typical sites of affection (skull, shoulder region, sternum, spine, femurs, tibia and spleen). The scale was set at a radiance of 10^5^ to10^6^ (F stop 1 autoexposure 10^4^ counts emission filter 620 nm). (D) A graph of the average luminescence [radiance (photons/second/cm^2^/steradian)] of BALB/c nu/nu male mice (n = 14) from day 0 to day 25. The luminescence value for each mouse is the average of ventral, dorsal and lateral (right/left) views. (E) A correlation plot of average luminescence for each mouse compared to their respective serum M315 values on day 10 (r = 0.9648, p<0.0001). Similar data were obtained on day 20. (F) DLIT and CT of a mouse with co-localization of tumor signal and the skeleton. The orange spheres depict where signal gradients are located. Pictures of various bones after removing skin or explanting organs, scales in photons/second/cm^2^/steradian, (G) spine, (H) ribs, (I) sternum, (J) femur and tibia, (K) kidneys, (L) lungs and heart, (M) spleen, (N) intestines and (O) liver.

To increase sensitivity of detection, MOPC315.BM.Luc cells were injected i.v. at a higher dose (5×10^6^) and into BALB/c nu/nu which lack hair and are thus better suited for BLI and the 3D bioluminescence imaging technique Diffuse Luminescent Imaging Tomography (DLIT). Immediately after injection, cells were clearly observed in the lung region in all mice injected. Most importantly, a weak signal, 5–10 fold higher than the corresponding region of the control mice, could be observed in the tibiofemoral region already 1h after injection (Fig. 3B). Such tibiofemoral signals were observed in 14 out of 14 mice, average photons detected were 1.3×10^5^±5.4×10^3^ SD versus 1.9×10^3^±2.4×10^2^ SD for non-injected controls (p<0.0001), indicating that tumor cells had entered the bone marrow immediately after injection. On day 2, the signal from the lungs had disappeared and a very weak signal was located in the spleen and the liver. By day 5, signals were found in multiple foci in bones, primarily the femurs, the lower part of the spine, and the hip. Tumor development was followed by BLI until the mice developed paraplegia (Fig. 3A,C,D) (average time for endpoint was 33 days, n = 14, Fig. S3A). As may be seen, the average luminescence signal decreased on day 2 and 5 to about 8% of immediate post-injection values, and recovered to initial levels between days 8 and 10 (Fig. 3D). This indicates that a substantial number, perhaps 90%, of cells may die in the days following i.v. injection. Progressive tumor growth was confirmed by increasing levels of myeloma protein in sera (Fig. S3B). Luminescence signals were highly correlated with serum M315 levels (Fig. 3E, r = 0.9648, p<0.0001). At onset of paraplegia, when the luminescence signal usually was strongest (see Fig. 3C for a typical example), DLIT and CT were performed on individual mice. Tumor tissue and the axial skeleton were co-localized in 3D (Fig. 3F). To confirm that the luminescence signal was in fact emanating from the skeleton, individual bones were dissected, or only skin was removed, before performing high resolution BLI, to more clearly expose skeletal sites (Fig. 3G–J). This procedure enhanced sensitivity, and individual foci could clearly be seen in spine (Fig. 3G), ribs (Fig. 3H), sternum (Fig. 3I) and femur and tibia (Fig. 3J). The strong signal emanating from the lower spine in most mice (e.g. Fig. 3A,C,F,G) is compatible with early occurrence of paraplegia due to compression of the spinal cord in diseased mice. In addition, internal organs were dissected and analyzed by BLI (Fig. 3K–O). A strong signal was observed from the spleen and much lower signals from other organs. Co-localization of tumor and axial skeleton is further demonstrated in two videos (full mouse in Fig. S4 and close-up in Fig. S5). Note that a strong signal corresponding to the spleen was observed, but no signals from other organs.

### MOPC315.BM Cause Disease Primarily in the Bone Marrow

**Figure 4:**
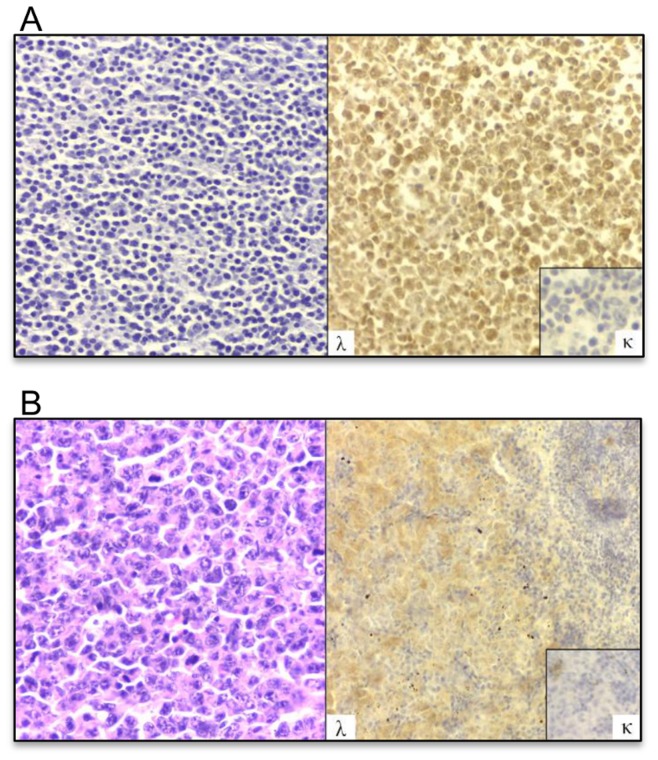
Histology and immunostaining of organs from paraplegic BALB/c mice injected with 5×10^5^ MOPC315.BM (IgAλ2^315^) cells i.v. (A) Bone marrow. Left: H&E staining. Right: Immunostaining with anti-λ or anti-κ (inset) antibodies, developed by HPRO (brown). (B) Spleen, red pulp. Left: H&E staining. Right: Immunostaining with anti-λ or anti-κ (inset) antibodies.

On autopsy, all paraplegic BALB/c mice injected with MOPC315.BM had visible tumor growth in spleens (a hematopoietic organ in the mouse), resulting in splenomegaly, but more rarely in the liver and other organs. It was macroscopically difficult to determine bone disease in paraplegic mice, even though visible tumors were sometimes seen penetrating the cortical bone. By microscopic analysis of H&E-stained sections, tumor growth was routinely found in bone marrow of femurs (Fig. 4A) and in spleens (Fig. 4B), as well as infrequently in the livers, fallopian tubes and subcutaneous tissues (Fig. S6). Immunohistochemistry demonstrated that MM cells expressed lambda and not kappa Ig L chains consistent with MOPC315 producing IgAλ2 myeloma protein (Fig. 4A,B).

### Fluorescent Protein (DsRed)-labeled MOPC315.BM Cells Preferentially Home to the Bone Marrow

**Figure 5:**
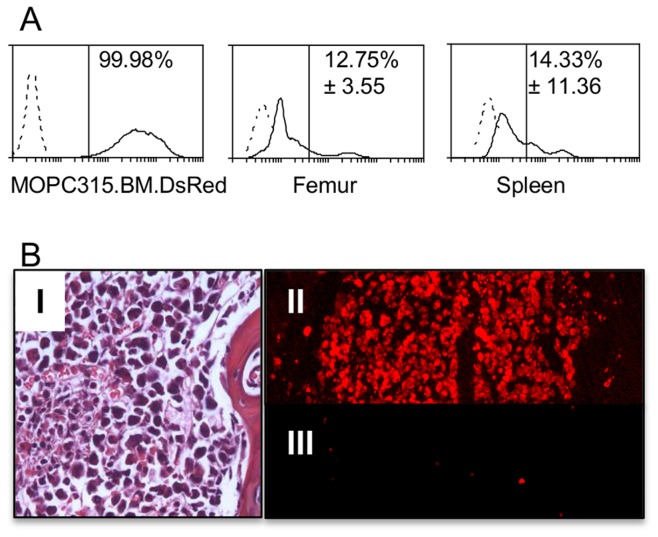
Flow cytometry and histology of MOPC315.BM DsRed cells injected i.v. in C.B-17 SCID (5×10^6^) and BALB/c mice (1×10^6^). (A) Upper left: Flow cytometric histograms of MOPC315.BM DsRed cells (solid line) and as a control MOPC315.BM cells (dashed line). Flow cytometry from a representative paraplegic C.B-17 SCID mouse. Upper right: Femur cells (solid line) and normal femur (dashed line). Lower right: spleen (solid line) and normal spleen (dashed line). The average and standard deviations for each organ are shown on each plot. Backgrounds in a non-injected mouse (to the right of the vertical line) were for femur 1.34% and spleen 1.68%. (B) I: H&E staining from a femur of a paraplegic BALB/c mouse. II: Fluorescence microscopy of sections of same femur. III: Femur from a mouse injected with non-fluorescent MOPC315.BM (control).

To confirm the presence of MOPC315.BM cells in the bone marrow and spleen, we transfected MOPC315.BM with DsRed, and injected cells i.v. into C.B-17 SCID mice. Upon onset of paraplegia, femurs and spleens were analyzed by flow cytometry (Fig. 5A) and femurs by immunohistochemistry (Fig. 5B) for the presence of MOPC315.BM.DsRed cells. For all injected mice (n = 3), DsRed positive cells were detected in bone marrow flushed from femurs. Cells were also detected in the spleen (Fig. 5A). MOPC315.BM.DsRed cells were present in the extravascular space of femur bone marrow, as shown by microscopy of H&E stained sections from paraffin-embedded tissue (Fig. 5B). These results essentially confirm the conclusions of Luciferase-based whole body imaging, and support a bone marrow-tropism of the MOPC315.BM cell line.

### MOPC315.BM.Luc Injected Mice Develop Osteolytic Lesions

**Figure 6:**
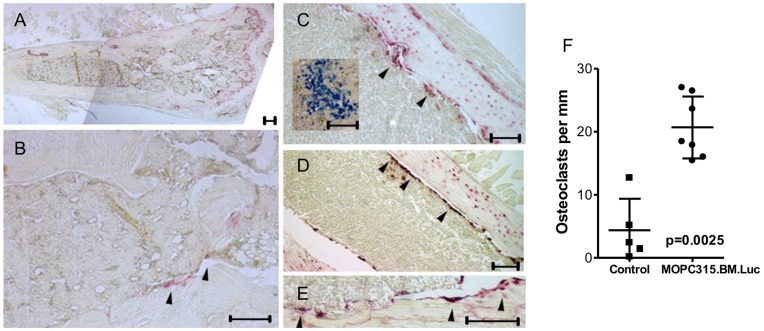
Osteoclasts in bone marrow of NSG mice infiltrated with MOPC315.BM.Luc. (A,B) Representative images of TRAP+ staining from the bone marrow of an age-matched control NSG mouse injected with RPMI 1640. (C–E) Representative images of TRAP+ staining from the bone marrow of an NSG animal injected with MOPC315.BM.Luc. Inset in (C) shows bone marrow localization of transplanted MOPC315.BM.Luc cells using staining for IgA. TRAP+ osteoclasts are indicated by black arrow heads. In all cases scale bar is 200 µm. (F) Quantification of osteoclasts per mm bone surface. Controls are age-matched RPMI-injected NSG mice.

**Figure 7:**
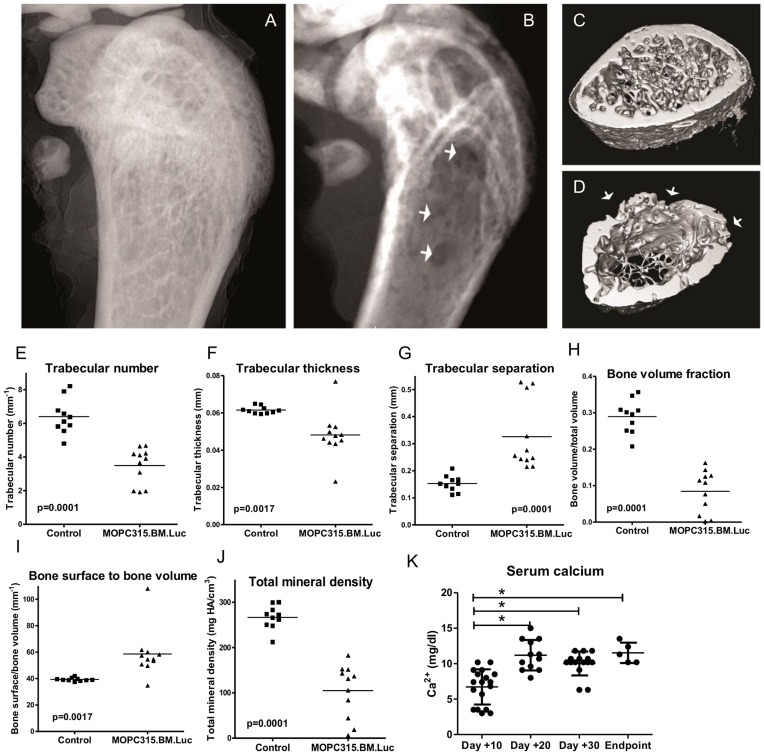
Osteolysis induced by MOPC315.BM.Luc cells. Transmission X-rays of distal femurs of NSG mice injected with either RPMI 1640 (A) or MOPC315.BM.Luc (expressing a doxycycline inducible non-functional control shRNA) (B) show extensive osteolytic lesions in the latter (white arrows). Similarly, µCT 3D reconstruction of the respective NSG femurs demonstrate reduced wall thickness (white arrows) and trabecular structures in bone marrow infiltrated by MOPC315.BM.Luc (expressing a doxycycline inducible non-functional control shRNA) (D) compared to age-matched controls (C). (E–J) Quantification of bone structure changes in BALB/c mice 5–8 weeks after injection of MOPC315.BM.Luc (5×10^5^ cells) and in age-matched controls. (K) Measurement of Ca^2+^ in serum of BALB/c mice at various time points after injection with MOPC315.BM. *p<0.002.

Osteolysis is a hallmark of human MM. Mice with growth of MOPC315.BM.Luc cells in bone marrow developed osteolytic lesions by several criteria. Firstly, MOPC315.BM.Luc induced an increased number of TRAP+ osteoclasts, compared to control mice injected with RPMI 1640 tissue culture medium (Fig. 6). Secondly, transmission X-rays demonstrated osteolytic lesions of femurs (Fig. 7A,B) and tibiae (data not shown), which were confirmed by µCT (Fig. 7C,D). In addition, severity of osteolytic lesions was quantified by bone structure analysis via µCT (Fig. 7E-J, Fig. S7). Osteolysis was evident in mice 5–8 weeks after injection of MOPC315.BM.Luc (Fig. 7E–J) as well as in mice with paraplegic disease (6–11 weeks after injection; Fig. S7). µCT assessment of total, trabecular and cortical bone compartments revealed highly significant changes in mice after injection of MOPC315.BM.Luc cells as compared to controls. Disturbed architecture of trabecular bone was demonstrated by decrease of trabecular number and trabecular thickness, and increase of trabecular separation (Fig. 7E–G, Fig. S7A–C). Overall bone volume decreased as evidenced by reduced bone volume fraction and an increased ratio of bone surface to bone volume (Fig. 7H–I, Fig. S7D–E). Moreover, the total mineral density was significantly reduced in MOPC315.BM.Luc injected mice (Fig. 7J, Fig. S7F). Thirdly, mice injected with MOPC315.BM had increased levels of calcium (Ca^2+^) in sera. For unknown reasons, a plateau level of Ca^2+^ was obtained by day 20 (Fig. 7K). Taken together, these results demonstrate that the MOPC315.BM cell line induces osteolytic lesions, a hallmark of human MM, when delivered by i.v. injection.

## Discussion

We here describe a novel variant of MOPC315, MOPC315.BM, that after i.v. injection of *in vitro*-cultured cells induce MM-like disease in the axial skeleton of syngeneic BALB/c mice, BALB/c nu/nu, C.B-17 SCID and NSG. Luciferase-labeled cells (MOPC315.BM.Luc) allow spatiotemporal resolution of bone disease development by repeated *in vivo* imaging. Injected mice develop osteolytic lesions, a hallmark of human MM. This novel mouse MM model could be useful for studies of bone marrow tropism, efficacy of drugs, mechanism of osteolysis, and immunotherapy. An important aspect of the current MOPC315.BM model is that experiments can be performed in an immunocompetent and readily available laboratory mouse strain, BALB/c. A further advantage is that the white fur of this albino strain makes *in vivo* imaging more sensitive and simpler to perform than in mice with black fur.

The results demonstrate that a mineral oil-induced plasmacytoma, MOPC315 (ATCC), adapted to *in vitro* growth, can cause paraplegia in about 80% of mice within 100 days after i.v injection. This finding was not anticipated since MOPCs are generally considered to be poor models of MM bone disease, even though they readily form extramedullary plasmacytomas after local injection. It may be that MOPC315 has an increased tropism for bone marrow compared to other MOPC lines and bone marrow tropism may thereby vary between different MOPC lines. The results further demonstrate that variants of the MOPC315 (ATCC) cell line can be obtained that more rapidly induces MM-like bone disease. Thus, a cell line selected for high tumor take by s.c. injection, MOPC315.4, caused paraplegia in all i.v.-injected mice within 65 days. After 9 cycles of i.v. injection of MOPC315.4 and recovery of cells from femurs of paraplegic mice, the MOPC315.BM cell line was obtained that following i.v. injection caused paraplegia in all mice within 35 days.

It is unclear whether the consecutive s.c. and i.v. selection procedures resulted in either a gradual change of phenotype or in a selection of rare cells with more MM-like features, pre-existing in the parental plasmacytoma cell line. The fact that decreasing amounts of injected cells were needed with progressive cycles could indicate that the *in vivo*-selection might have enriched for a pre-existing variant. It is also unknown whether preferential growth in bone marrow was due to increased homing to the bone, or if cells simply grew better once they had settled in the bone marrow microenvironment or both.

MOPC315.BM cells labeled with firefly luciferase (MOPC315.BM.Luc) could be followed by repeated bioluminescent imaging of i.v.-injected mice. Overall, the results were similar in normal BALB/c and T cell-deficient BALB/c nu/nu mice. However, sensitivity was clearly higher in furless BALB/c nu/nu mice, the nude strain being the recommended model for DLIT (Living Image® User Manual, Caliper Life Sciences). Sensitivity was further increased by injection of a higher number of cells. Immediately after injection, cells were found primarily in the lung, but also in the spleen and the liver. However, and importantly, a minor fraction of cells were found in the tibiofemoral region already 1 h after injection. Early invasion of bone marrow is consistent with results obtained with i.v. injection of ^51^Cr-labeled 5T2MM cells [37]. (However, note that detection of luciferase-labeled cells by BLI ensures that cells are alive while a detection of a ^51^Cr signal does not necessarily guarantee the presence of living cells). On day 5, progressively growing MM foci were seen in multiple sites of the axial skeleton, with signals first being detected in the femurs and the spine. The spleen signal progressed with time, while lung and liver signals decreased. Affection of other organs was only infrequently detected. MM growth in spleen is consistent with extramedullary hematopoiesis in this organ, and is also found in the 5TMM models [12], [14].

A considerable number of the injected cells apparently died since the total bioluminescence decreased by >90% on day 2, and only recovered to initial post-injection values after day 8. Since injection of about 1000 MOPC315.BM cells caused paraplegia in about 50% of mice, it may be roughly extrapolated that perhaps only 100 MOPC315.BM cells, surviving the days following i.v. injection, is sufficient to cause development of MM-like bone disease.

Even though relatively few bone foci were seen in BALB/c mice, the increased sensitivity in nude mice, combined with *ex vivo* imaging at endpoint, revealed that MM growth was more diffuse with multiple foci in single ribs and sternum. Consistent with this, DsRed-labeled MOPC315.BM cells were detected in every flushed femur of paraplegic mice. The average is consistent with that often observed in human MM (>10% malignant plasma cells) though we only measured three paraplegic mice. Note that the endpoint, paraplegia due to compression of the lower part of the spinal cord, is observed relatively early in mice injected with MOPC315.BM. Thus, in absence of paraplegia, much higher numbers of MM cells in bone marrow of femur might have been reached. Flow cytometry data show that the infiltration of MOPC315.BM cells in tibiae equals that of vertebral bodies (data not shown), however BLI data indicate that the lower vertebrae could have a higher density of MM cell foci than other bone sites, predisposing for paraplegia.

A hallmark of human MM is osteolytic lesions. The MOPC315.BM induced osteolytic lesions were detected by histology (staining for TRAP+ osteoclasts), transmission X-rays and µCT of isolated bones, and elevated Ca^2+^ levels in serum. Thereby, *ex vivo* µCT analysis of distal femurs demonstrated highly significant changes in trabecular and cortical architecture in MOPC315.BM injected mice compared to controls. However, the osteolytic lesions were difficult to detect by standard X-rays of living mice. Again, if mice could have been observed beyond development of paraplegia, osteolytic lesions might have become more pronounced.

Lynch and co-workers have previously described that MOPC315 grown in diffusion chambers i.p. have a preferential loss of cells which are of plasmacytoid appearance, resulting in enrichment of cells which secrete less myeloma protein, but have increased expression of surface BCR [38]. They have also described a variant, MOPC315^S^, that when injected i.v. readily gave rise to splenic tumor foci [39]. The bone marrow-homing MOPC315.BM appears distinct from these previously described variants since its production of secreted myeloma protein and BCR is similar to that of MOPC315.4, and since it has tropism for bone in addition to spleen.

It is generally believed that MM cells represent malignant counterparts of plasma cells that at the earlier B cell stage have been through a germinal center reaction[40]–[42]. However, it is unclear where the neoplastic process initially takes place. One possibility is that MM originates from plasma cells malignantly transformed within the bone marrow, and that MM cells later metastasize to other bones. Another possibility is that neoplastic cells originate in an extramedullary site, and then seed multiple bones where they are exposed to a microenvironment conducive to growth. Several pieces of evidence support the latter possibility. Firstly, MM has been associated with less differentiated clonogenic precursors found in blood[43]–[45]. Secondly, extramedullary plasmacytomas in humans can metastasize to bone [46]. Thirdly, ileocecal plasmacytoma in an aged gonadectomized mouse [31], as well as transplanted MOPC tumors [32], [33], can metastasize to the bone marrow. The present data are compatible with the idea that MM could originate from cells undergoing malignant transformation outside the bone marrow and that variants with increased bone marrow tropism seed multiple bones.

It is of great interest to find out why MM cells home to and grow in the bone marrow. A comparison of MOPC315.BM and MOPC315.4 at the protein and gene expression levels might offer clues as to why the former variant more rapidly causes bone disease. A number of factors could contribute, such as CD44 splice variants, chemokine and cytokine receptors, integrins, metalloproteases, and responsiveness to growth signals in the bone marrow microenvironment, including cytokines like IL-6 [47]. In a global approach to this issue, repeated and independent gene expression profiling of MOPC315.BM and MOPC315.4, and extensive flow cytometric and cytokine secretion analysis, has revealed stable differences (H. C. Jodal et al., proposals for further investigation). In addition, since the MOPC315.BM model was established in the BALB/c strain it might be possible, by use of available knock-out mice on this background, to study the influence of genes on MM cell homing and growth in the bone marrow.

In summary, MOPC315.BM and the 5TMM tumors [8], [12], [14] appear to share the ability to home to the bone marrow and cause osteolytic lesions. Similar to the 5TGM1 cell line [18], MOPC315 can be cultured *in vitro*, injected i.v., and causes MM-like disease. However, the MOPC315.BM has certain advantages for experimental manipulation since we here show that it may be readily labeled by luciferase or DsRed for tracking by *in vivo* imaging, and since it reproducibly causes bone disease in a common strain of laboratory mice, BALB/c. The model should be valuable for studying mechanisms of homing of MM cells to bone marrow and drug treatment. Moreover, since so much is known about idiotype-specific T cell immunotherapy of s.c. extramedullary MOPC315 plasmacytomas[26]–[30], the present model should allow the application of T cell immunotherapy to MM cells in the bone marrow (Hofgaard and Bogen, proposals for further investigation).

## Materials and Methods

### Ethics Statement

This study was carried out in accordance with the 3Rs principle. Experiments were approved by the National Committee for Animal Experiments (Oslo, Norway), application no. 2153. All surgery and euthanization was performed under isoflurane (2.5–5% inhalation) or midazolam and fentanyl (both 5 mg/ml, i.p. injection) anesthesia, and all efforts were made to minimize suffering.

### Mice

BALB/c, BALB/c nu/nu and C.B-17 SCID mice were obtained from Taconic (Ry, Denmark). NOD scid gamma (NSG) were obtained from Charles River (Germany).

### The MOPC315.BM Cell Line

MOPC315 [48] was obtained as an *in vitro*-adapted cell line from ATCC (Manassas, Virginia). This cell line was cycled between *in vitro* culture and *in vivo* growth s.c.; the resulting cell line, MOPC315.4, has been used in our previous s.c. tumor challenge experiments [26], [27], [34], [49]. To obtain a variant of MOPC315 that homes to the bone marrow and grows in that site, a large number of MOPC315.4 cells (2×10^6^) were injected i.v. into five BALB/c mice. The first mouse to develop paraplegia was euthanized, bone marrow cells were flushed from the femurs, and cultured in RPMI 1640 GlutaMAX (Gibco) supplemented with 1% MEM NEAA 100x (Gibco), 1% sodium pyruvate (Gibco), 0.005% 1M I-thioglycerol (Sigma) solution, 0.03% Gensumycin 40 mg/ml (Sanofi aventis) and 10% fetal bovine serum (Biochrom AG). Normal bone marrow cells eventually died and the culture was taken over by expanding MOPC315.4 cells, which were expanded and injected i.v. into new BALB/c mice. With consecutive passages, mice developed paraplegia more rapidly, and the number of MOPC315.4 cells injected was eventually reduced to 2.5×10^5^. After the third and the ninth passages, MOPC315.4 was cloned *in vitro* by limiting dilution, and clones that grew well and produced M315 myeloma protein were selected for further use. After 9 passages, the resulting cell line, MOPC315.BM, was frozen in aliquots.

### Labeling of MOPC315.BM with Luciferase and DsRed

Luciferase labeling: MOPC315.BM cells were co-transfected by electroporation with the pGL3-Control vector containing the luciferase gene (Promega), and the pcDNA 3.1(+) vector containing the gene for neomycin resistance (Invitrogen). For DsRed labeling, MOPC315.BM cells were co-transfected with the pCMV-DsRed-Express vector (Clontech), and the pcDNA 3.1(+) vector. Clones with high expression were re-cloned by limiting dilution, selected for high expression, frozen in aliquots, and used for tumor challenge experiments.

### Tumor Challenges. Measurement of Myeloma Protein

MOPC315 (ATCC), MOPC315.4, MOPC315.BM, MOPC315.BM.Luc, and MOPC315.BM.DsRed cells were cultured *in vitro* (37°C, 5% CO_2_) and harvested when growing exponentially, then centrifuged (300 g, 7 min) and resuspended in PBS (Gibco-BRL, Carlsbad CA). Cells were counted and injected i.v. in the tail vein. Injection dosage used is indicated in the figure legend or text. Endpoints were 1) paraplegia, 2) visible extramedullary tumor disease with tumor size >1 cm in diameter, 3) distended abdomens, or 4) weight loss >10%. The mice were sacrificed on the same day the endpoint was reached. Disease was verified by measurement of M315 myeloma protein in ELISA [26].

### Whole Body in vivo Imaging of Tumor Cells

Imaging of luciferase activity *in vivo* was performed essentially as described [36]. Mice were injected i.p. with D-luciferin (150 mg/kg body weight) and imaged using either the IVIS Spectrum or IVIS Spectrum CT imaging systems (Caliper Life Sciences, Hopkinton, Massachusetts). Images were acquired from 10 to 20 minutes post substrate injection. Data were analyzed using LivingImage® (Caliper Life Sciences) software. Each image included a non-tumor bearing control mouse also administered D-luciferin. Luminescence for each side or region of tumor bearing mice was quantified using average photons/second/cm^2^/steradian, with the respective area on the non-tumor bearing control mice subtracted. Diffuse Light Imaging Tomography (DLIT) and Computer Tomography (CT) modes were performed on an IVIS Spectrum CT (Caliper Life Sciences). For the DLIT mode, four spectral images were acquired at 580 nm, 600 nm, 620 nm, and 640 nm. Auto exposure was set at 10,000 photons. Sequence acquisition was performed between 5 and 40 minutes post i.p. injection of D-luciferin. For CT, the standard one mouse or medium resolution settings were used.

To increase sensitivity of detection of MOPC315.BM.Luc, BALB/c nu/nu mice, which lack hair and are thus better suited for BLI and the 3D bioluminescence imaging technique DLIT, were used in some experiments (Fig. 3). However, BALB/c nu/nu mice also lack a thymus and thus T cells, which may affect the immune response and tumor growth.

### Flow Cytometry

DsRed is, in contrast to luciferase, immunogenic in BALB/c mice, and immunodeficient mice thus have to be used when injecting DsRed-transfected cells. C.B-17 SCID mice lack both B and T cells due to a defect in V(D)J recombination. This may affect tumor growth to some degree as an immune response towards the tumor cells will be lacking. C.B-17 SCID mice i.v. injected with MOPC315.BM DsRed cells were followed until endpoint and then euthanized. Bone marrow was flushed with complete medium, using a needle and syringe, from femurs. Spleens were dissected from individual mice and placed in complete medium. Spleens were then minced with a scissor and gently pushed through a sieve (Sigma). The cell suspensions were centrifuged (300 g 7 min), resuspended in erythrocyte lysis solution (TRIS 17 mM, NH_4_Cl 140 mM, pH 7.2), then washed with PBS and thereupon fixed (1% paraformaldehyde (PFA)). Bone marrow was gently pipetted to obtain a cell suspension, centrifuged, then resuspended in PBS with 5% BSA, and fixed in PFA. The samples were run and analyzed on the FL2 channel of a FACSCalibur flow cytometer using Cell Quest Pro™ and WEASEL softwares. To determine the percentage of tumor cells in a sample, a marker was placed in front of the negative controls (femur and spleen cells from a normal mouse) such that >98% of the cells were to the left of that marker. Using this marker, all cells to the right were judged to be positive. The mean and standard deviation from three mice were then calculated. The backgrounds in the negative controls (percentage of the cells to the right of the marker) are indicated in the figure legend.

### Histochemistry and Microscopy

When MOPC315.BM-injected BALB/c mice developed paraplegia, samples were taken from limbs, liver, spleen, lungs, gastrointestinal tract, kidneys and heart, and fixed in 4% PFA before embedding in paraffin. 5-µm sections throughout the organs were made with a rotation microtome (Leica RM2135). One slide was stained with haematoxylin and eosin and two additional slides were immunostained for light chain kappa and lambda in order to visualise the MOPC315.BM cells. For immunostaining, the endogenous peroxydase was quenched by incubating the slides with a 50-mL methanol, 0.5-mL H_2_O_2_ (3%) (Merck, Darmstadt, Germany) solution for 20 min. Autoclave heating was used for antigen retrieval by immersing the slides in a autoclavable tray, containing 5 mL of Target Retrieval Solution (Dako, Glostrup, Denmark) and 45 mL of deionised water and autoclaving at 120°C for 10 minutes. Slides were blocked using a blocking buffer (Universal Blocking Reagent, Bio Genex) to reduce non-specific background staining. The horseradish peroxydase coupled anti-mouse lambda and kappa antibodies (Rockland, Gilbertsville, PA, USA) were incubated for 2 h at room temperature at a dilution of 1∶1000. Chromogenic visualisation was accomplished through the use of a DAB reaction (3,30-diaminobenzidine hydrochloride kit; (DAB), Dako), followed by counterstaining with haematoxylin.

For immunohistochemistry related to Fig. 6, the tissue was fixed in 4% PFA, decalcified in 10% EDTA (pH 7.4), embedded in paraffin before being sectioned (4 µm) and rehydrated. After HEIR (5 mM citrate buffer pH 7), CD138 mAb (Clone 281–2, BD Pharmingen) and an AP labeled anti-IgA (Sigma, St. Louis, Missouri) were used to identify MOPC315.BM.Luc cell infiltration. All sections were analyzed using a Nikon Eclipse E 800, and digital images processed with AnalySIS Soft Imaging System, or images were captured using a Nikon Eclipse TE 2000-U with either a DS-Qi1Mc (5.03) or Dxm1200 digital camera, with NIS-Elements BR version 3.0 software.

TRAP staining was done according to Acid Phosphatase, Leukocyte (TRAP) Kit from Sigma (St. Louis, Missouri), without HEIR. These sections were then photographed, regions of bone marked and the osteoclasts counted by three individuals, then averaged and divided by bone surface length (mm) (Fig. 6F).

For detection of MOPC315.BM.DsRed cells, femurs were fixed in 4% PFA, decalcinated, embedded in paraffin, and de-waxed. Sections were analyzed by fluorescence microscopy (Nikon Eclipse E 800) or H&E stained and analyzed by light microscopy (Leitz Dialux), and digital images processed with AnalySIS Soft Imaging System.

### Transmission X-ray and µCT

NSG mice were injected with a MOPC315.BM.Luc clone which expresses a doxycycline inducible non-functional control shRNA (Fig. 7A–D). Explanted bones were fixed in 4% PFA and X-rayed in an experimental µCT facility[50]–[51] (Fraunhofer Development Center for X-ray Technology EZRT, Fürth, and Institute for X-ray Microscopy, University Würzburg, Germany). X-ray source from Excillum, using a liquid metal jet (Galinstan, GaInSn alloy) anode with a 15 µm spot size at 70 kV and 80 Watt. Images were detected using a Gadolinium oxysulphide scintillator and a CMOS camera with 6.5 µm pixel size. Isosurface from µCT data was computed using Image J (version 1.47c) and the BoneJ plugin (version 1.34) [52].

For bone structure analysis, BALB/c mice were injected with MOPC315.BM.Luc (Fig. 7E–J, Fig. S7). Total femur length was measured using digital calipers on freshly isolated bones. At the distal femur, endocortical contours were drawn for a region with a height equivalent to 5% of the femur length, beginning at 850 µM from the growth plate and proceeding proximal. Thresholds of 1891 mg hydroxyapatite (HA)/cm^3^ were used for distal femur that is rich in trabecular bone. Total mineral density (TMD), bone volume, total volume, bone surface, trabecular number, separation and thickness were studied on excised samples using a desktop µCT 40 (Scanco Medical AG, Brüttisellen, Switzerland). Femurs were scanned and images were reconstructed to an isotropic voxel size of 10.5 µm. All quantitative analyses were performed with the system’s software (Scanco Eval 6.5–1).

### Measurement of Serum Ca^2+^

Serum Ca^2+^ was measured using the Calcium Colorimetric Kit Assay from BioVision (Milpitas, California).

### Statistics

The logrank test was used to compare the Kaplan-Meyer survival plots. Two-tailed Mann-Whitney was used to compare groups for levels of serum myeloma protein, bioluminescent radiance, serum Ca^2+^, bone surface osteoclasts as evaluated by histology and all quantitative data concerning the µCT and X-rays. Correlation between levels of M315 antigen and whole body bioluminescence was determined on the basis of the Spearman's correlation coefficient.

## Supporting Information

Figure S1 **Serum concentrations of M315 from tumor challenge studies.** (A) Shows the same data as in Fig. 1C, but displayed with mean and SD for each group at the different time points. Comparison of i.v. injection of 2×10^5^ cells of either MOPC315 (ATCC), MOPC315.4 or MOPC315.BM. n = 12/group. (B) Shows the same data as in Fig. 2B, but displayed with mean and SD for each group at the different time points. Comparison of i.v. injection of 2×10^5^ cells of MOPC315.BM.Luc (n = 13) overlaid the data of MOPC315.BM (n = 12) from Fig. S1A.
(TIF)

Figure S2 **Effect of bortezomib on MOPC315.BM cells tested** ***in vitro*** **in an MTT assay.**
(TIF)

Figure S3 **Growth of MOPC315.BM.Luc cells in BALB/c nu/nu mice.** (A) Tumor take experiment. (B) M315 concentration in ng/ml (solid lines of individual mice). Lower detection limit of 10 ng/ml.
(TIF)

Figure S4 **Video of DLIT and CT of a whole BALB/c nu/nu mouse.** Shows the co-localization of MOPC315.BM.Luc and the axial skeleton. Note the presence of tumor cells in the spleen, but no other organs.
(AVI)

Figure S5 **Close-up video of DLIT and CT of a BALB/c nu/nu mouse.** Shows the co-localization of MOPC315.BM.Luc and the axial skeleton, as shown as a whole mouse in Fig. S4. Note the presence of tumor cells in the spleen, but no other organs.
(AVI)

Figure S6 **Infrequent examples of microscopically detected MOPC315.BM tumors.** As shown in Fig. 2 in paper version, spleen and bone marrow were almost always affected, while affection of other organs was rarer. Shown are infrequent examples of microscopic growth in liver (A) and fallopian tube (B), and subcutaneous macroscopic growth (C).
(TIF)

Figure S7 **Osteolysis induced by MOPC315.BM.Luc at paraplegia.** BALB/c mice were injected with MOPC315.BM.Luc (2×10^5^ cells) i.v. and distal femurs were analyzed by µCT at paraplegia (6–11 weeks after injection). Non-injected 10–11 weeks old BALB/c mice served as controls.
(TIF)

Table S1 ***In vitro*** **growth rate and** ***in vitro*** **M315 secretion.** All cell lines were cultured as previously described in this article. Cells growing exponentially were then harvested, counted, resuspended in medium and kept on ice until use. *In vitro* growth was estimated by an assay where 8 parallel samples of 50 µl at a concentration of 1×10^5^cells/ml (5000 cells) of a cell line was added to 200 µl of warm medium (37°C) in 8 wells of a 96 well, flat bottom Costar® plate (Corning Inc., USA). After standard incubation (37°C, 5% CO_2_) for 48 hours, and while the cultures were growing exponentially, the dish was placed on ice and the cells in each well were counted. The website (http://www.doubling-time.com/compute.php) was used to calculate doubling times. *In vitro* M315 secretion was measured by adding 3 parallel samples of 50 µl at a concentration of 1×10^8^cells/ml (5×10^6^cells) of a cell line to 200 µl of warmed medium (37°C) in 3 wells of a 24 well, flat bottom Costar® plate and incubated for 1 hour under standard conditions. The cultures were then immediately centrifugated and supernantants were stored at −70°C. For each well, the M315 concentration was determined from the mean of two independent M315 ELISAs. The average secretion rate was obtained using the added cell number (5×10^6^). The unpaired t test was used to calculate p values. *p<0.05 compared to MOPC315 (ATCC) and MOPC315.4. ^¶^Significantly higher than the other cell lines p<0.05.
(PPT)
