# Supplementary figures and images for: A Novel Mouse Model for Multiple Myeloma (MOPC315.BM) That Allows Noninvasive Spatiotemporal Detection of Osteolytic Disease
Source: PLoS One. 2012 Dec 20;7(12):e51892. doi: 10.1371/journal.pone.0051892 (PMC3527494; doi:10.1371/journal.pone.0051892)

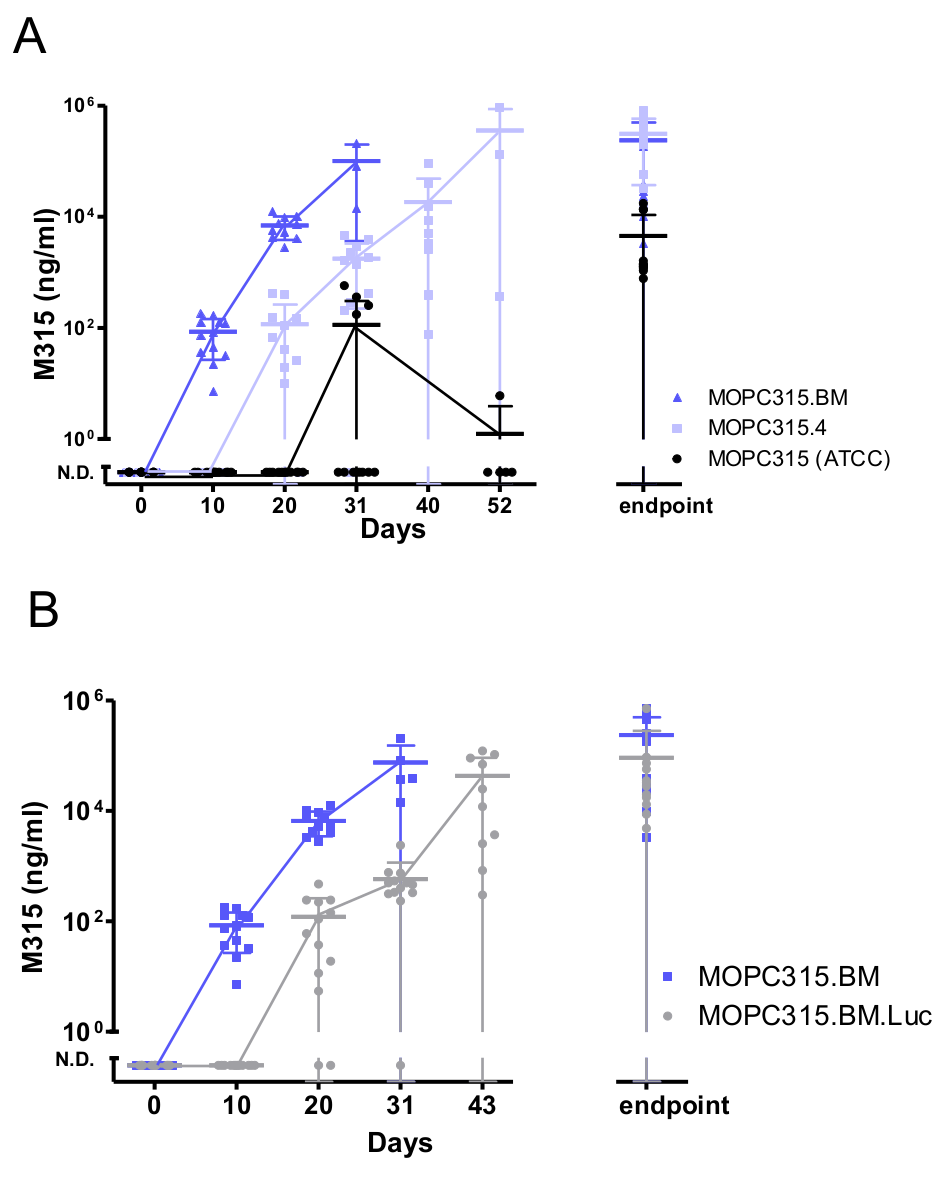

Supplement: Figure S1 — Serum concentrations of M315 from tumor challenge studies. (A) Shows the same data as in Fig. 1C, but displayed with mean and SD for each group at the different time points. Comparison of i.v. injection of 2×10^5^ cells of either MOPC315 (ATCC), MOPC315.4 or MOPC315.BM. n = 12/group. (B) Shows the same data as in Fig. 2B, but displayed with mean and SD for each group at the different time points. Comparison of i.v. injection of 2×10^5^ cells of MOPC315.BM.Luc (n = 13) overlaid the data of MOPC315.BM (n = 12) from Fig. S1A. (TIF) [file pone.0051892.s001.tif]

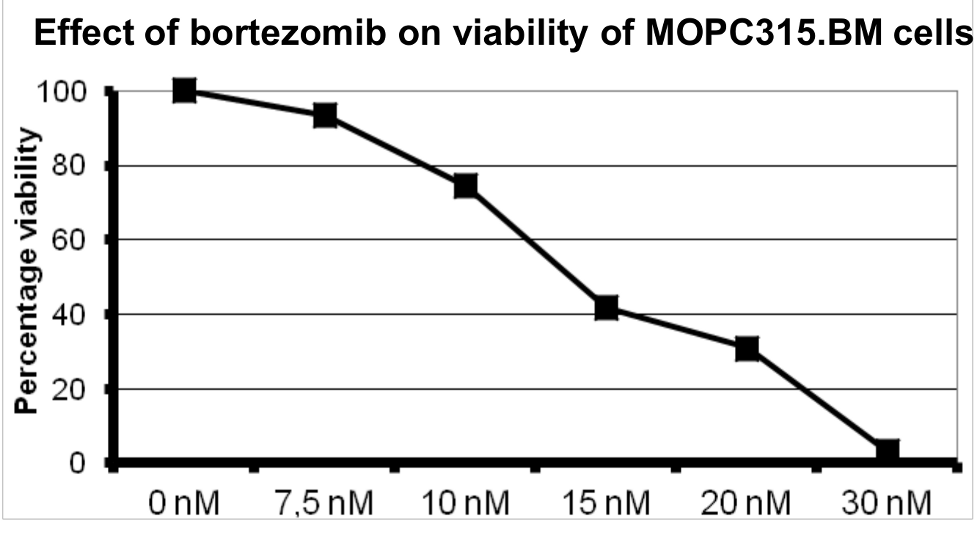

Supplement: Figure S2 — Effect of bortezomib on MOPC315.BM cells tested in vitro in an MTT assay. (TIF) [file pone.0051892.s002.tif]

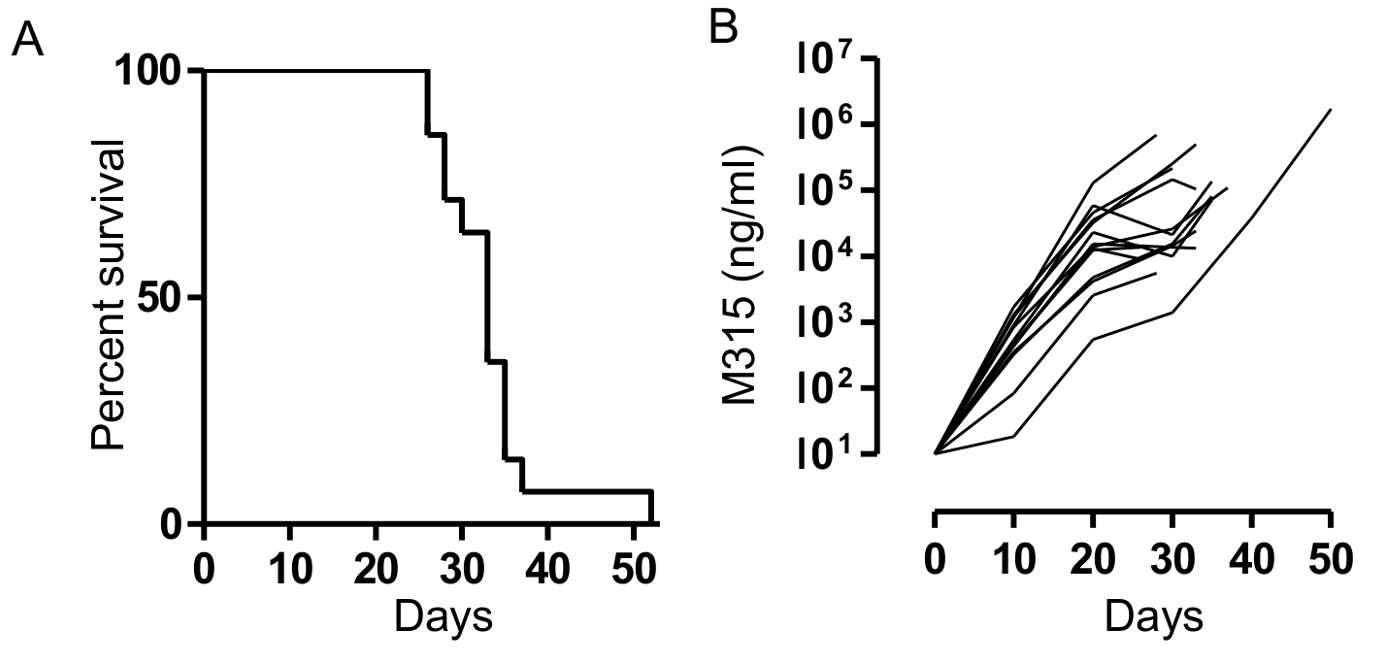

Supplement: Figure S3 — Growth of MOPC315.BM.Luc cells in BALB/c nu/nu mice. (A) Tumor take experiment. (B) M315 concentration in ng/ml (solid lines of individual mice). Lower detection limit of 10 ng/ml. (TIF) [file pone.0051892.s003.tif]

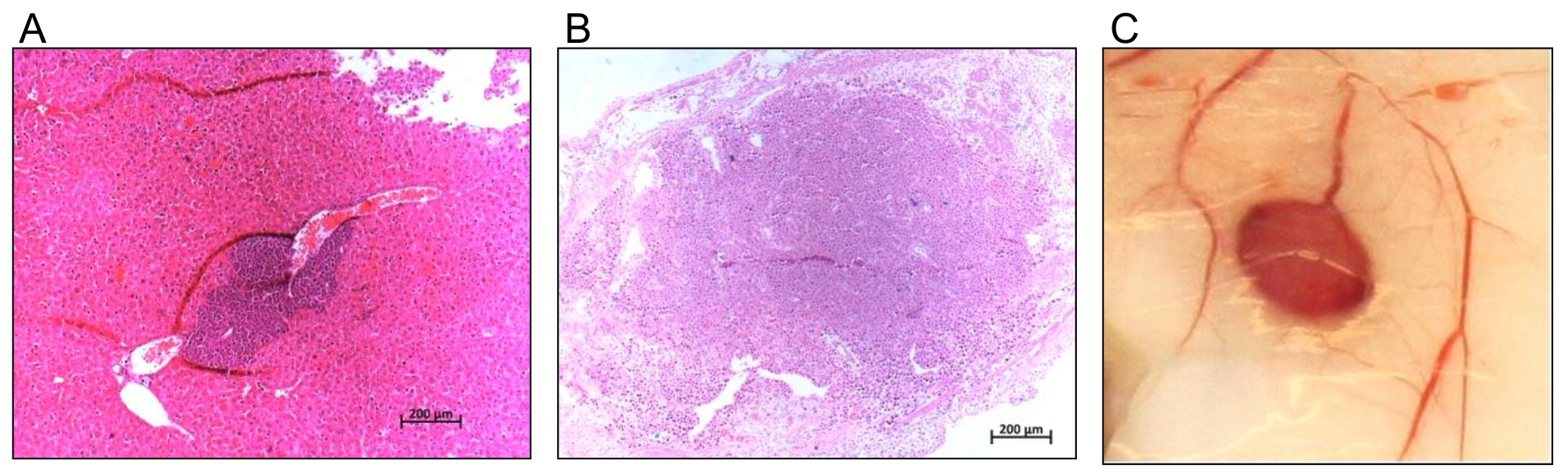

Supplement: Figure S6 — Infrequent examples of microscopically detected MOPC315.BM tumors. As shown in Fig. 2 in paper version, spleen and bone marrow were almost always affected, while affection of other organs was rarer. Shown are infrequent examples of microscopic growth in liver (A) and fallopian tube (B), and subcutaneous macroscopic growth (C). (TIF) [file pone.0051892.s006.tif]

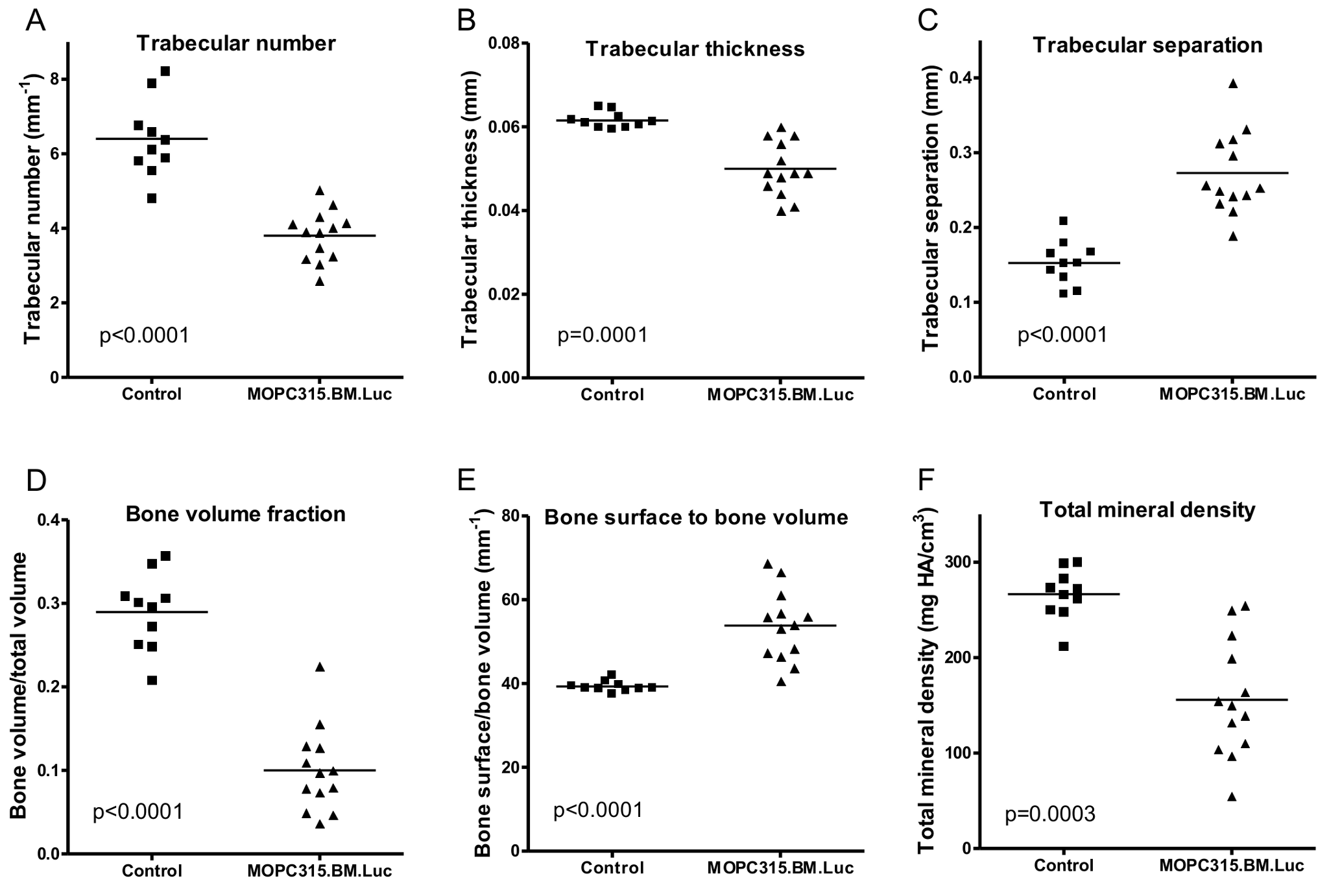

Supplement: Figure S7 — Osteolysis induced by MOPC315.BM.Luc at paraplegia. BALB/c mice were injected with MOPC315.BM.Luc (2×10^5^ cells) i.v. and distal femurs were analyzed by µCT at paraplegia (6–11 weeks after injection). Non-injected 10–11 weeks old BALB/c mice served as controls. (TIF) [file pone.0051892.s007.tif]

## Slide 1
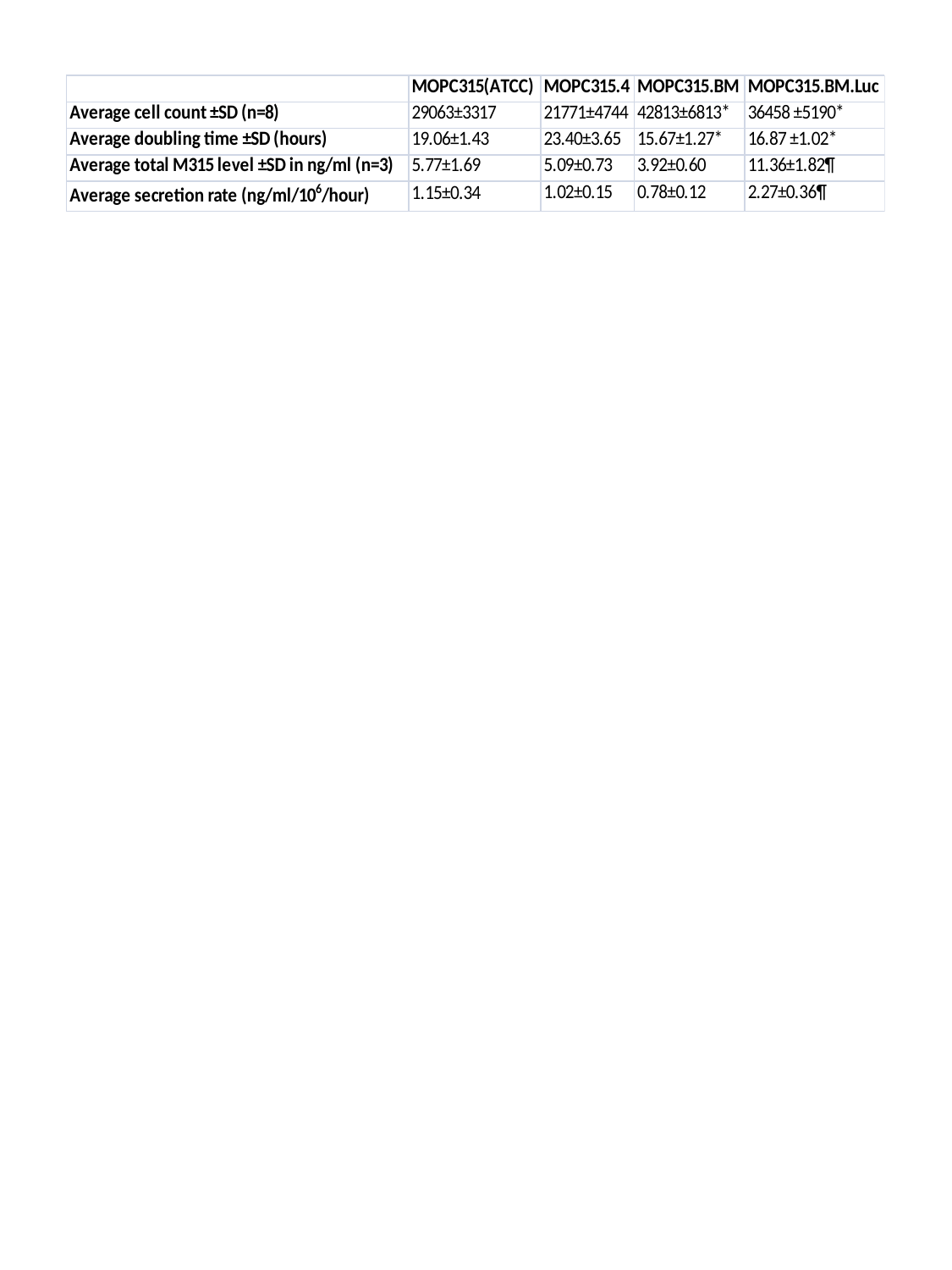

Supplement: Table S1 — In vitro growth rate and in vitro M315 secretion. All cell lines were cultured as previously described in this article. Cells growing exponentially were then harvested, counted, resuspended in medium and kept on ice until use. In vitro growth was estimated by an assay where 8 parallel samples of 50 µl at a concentration of 1×10^5^cells/ml (5000 cells) of a cell line was added to 200 µl of warm medium (37°C) in 8 wells of a 96 well, flat bottom Costar® plate (Corning Inc., USA). After standard incubation (37°C, 5% CO2) for 48 hours, and while the cultures were growing exponentially, the dish was placed on ice and the cells in each well were counted. The website (http://www.doubling-time.com/compute.php) was used to calculate doubling times. In vitro M315 secretion was measured by adding 3 parallel samples of 50 µl at a concentration of 1×10^8^cells/ml (5×10^6^cells) of a cell line to 200 µl of warmed medium (37°C) in 3 wells of a 24 well, flat bottom Costar® plate and incubated for 1 hour under standard conditions. The cultures were then immediately centrifugated and supernantants were stored at −70°C. For each well, the M315 concentration was determined from the mean of two independent M315 ELISAs. The average secretion rate was obtained using the added cell number (5×10^6^). The unpaired t test was used to calculate p values. *p<0.05 compared to MOPC315 (ATCC) and MOPC315.4. ¶Significantly higher than the other cell lines p<0.05. (PPT) [file pone.0051892.s008.ppt]
